# Occupational class differences in diagnostic-specific sickness absence: a register-based study in the Finnish population, 2005–2014

**DOI:** 10.1186/s12889-017-4674-0

**Published:** 2017-08-22

**Authors:** Johanna Pekkala, Jenni Blomgren, Olli Pietiläinen, Eero Lahelma, Ossi Rahkonen

**Affiliations:** 1 Department of Public Health, University of Helsinki, P.O. Box 20 (Tukholmankatu 8B), 00014 Helsinki, Finland; 2 The Social Insurance Institution of Finland, P.O. Box 450, (Nordenskiöldinkatu 12), FIN-00056 KELA, Helsinki, Finland

**Keywords:** Sickness absence, Occupational class, Musculoskeletal diseases, Mental disorders, Changes

## Abstract

**Background:**

Musculoskeletal diseases and mental disorders are major causes of long-term sickness absence in Western countries. Although sickness absence is generally more common in lower occupational classes, little is known about class differences in diagnostic-specific absence over time. Focusing on Finland during 2005–2014, we therefore set out to examine the magnitude of and changes in absolute and relative occupational class differences in long-term sickness absence due to major diagnostic causes.

**Methods:**

A 70-per-cent random sample of Finns aged 25–64 linked to register data on medically certified sickness absence (of over 10 working days) in 2005–2014 was retrieved from the Social Insurance Institution of Finland. Information on occupational class was obtained from Statistics Finland and linked to the data. The study focused on female (*n* = 658,148–694,142) and male (*n* = 604,715–642,922) upper and lower non-manual employees and manual workers. The age-standardised prevalence, the Slope Index of Inequality (SII) and the Relative Index of Inequality (RII) were calculated for each study year to facilitate examination of the class differences.

**Results:**

The prevalence of each diagnostic cause of sickness absence declined during the study period, the most common causes being musculoskeletal diseases, mental disorders and injuries. The prevalence of other causes under scrutiny was less than 1 % annually. By far the largest absolute and relative differences were in musculoskeletal diseases among both women and men. Moreover, the absolute differences in both genders (*p* < 0.0001) and the relative differences in men (*p* < 0.0001) narrowed over time as the prevalence declined most among manual workers. Both genders showed modest and stable occupational class differences in mental disorders. In the case of injuries, no major changes occurred in absolute differences but relative differences narrowed over time in men (*p* < 0.0001) due to a strong decline in prevalence among manual workers. Class differences in the other studied diagnostic causes under scrutiny appeared negligible.

**Conclusions:**

By far the largest occupational class differences in long-term sickness absence concerned musculoskeletal diseases, followed by injuries. The results highlight potential targets for preventive measures aimed at reducing sickness absence and narrowing class differences in the future.

## Background

Long-term sickness absence is a major social, economic and health problem. It accounts for the majority of the costs of all sickness absence [1] and reflects ill health [2]. The most common diagnostic causes of long-term sickness absence are musculoskeletal diseases and mental disorders [1, 3–5]. Sickness absence due to mental disorders increased from the 1990s until the early 2000s [3–5], but a downward trend has been reported since the mid-2000s [5, 6]. There has been an equivalent trend in sickness absence due to musculoskeletal diseases in Finland [5], whereas sickness absence due to injuries, for instance, has remained relatively stable over time [5]. Previous studies have shown that those in lower occupational classes have more sickness absence (see, for instance) [7–10], and that class differences have persisted over time [11–14]. However, little is known about changes over time regarding occupational class differences in sickness absence attributable to different diagnostic causes.

Previous studies examining diagnostic-specific sickness absence have shown hierarchical occupational class differences in work-injury-related absences [15–18] and absence attributable to different somatic causes such as musculoskeletal diseases [16, 19–21], respiratory diseases [16, 22] and digestive disorders [15]. With regard to cardiovascular diseases, some studies report class differences only among men [15, 23]. Previous findings on mental disorders are mixed [15, 16, 24], and the results differing between specific diagnoses [25]. However, only few studies have examined occupational class differences in sickness absence simultaneously across several diagnostic causes. A British study [15] reported particularly large differences in long (7+ days) periods of sickness absence related to musculoskeletal diseases, injuries and respiratory diseases, and diagnosed mental disorders, such as depression and anxiety. A French study [16], in turn, revealed large occupational class differences in sickness absence related to musculoskeletal diseases and, among men, injuries, but the class differences were less profound in the case of mental disorders among both women and men.

Various factors have been shown to influence occupational class differences in sickness absence. Occupational class is a key indicator of socioeconomic position and implies, for instance, differences in physical and psychosocial working conditions across the occupational class hierarchy [26]. Previously, different work-related factors, in particular deleterious physical working conditions, have been shown to explain a major part of the observed hierarchical occupational class differences in sickness absence [7, 9, 10]. A French study [16], examining the contribution of work-related factors with occupational class differences in diagnostic-specific sickness, found that both adverse physical and psychosocial work-related factors were associated with the class differences in sickness absence due to musculoskeletal diseases. The class differences in sickness absence attributable to mental disorders were mainly related to work-stress factors, in particular low decision latitude and low social support, and adverse physical working conditions explained the occupational class gradient in sickness absence due to injuries [16]. Other main explanations for the occupational class differences in sickness absence have been shown to relate to health behaviours, such as smoking, alcohol consumption, weight and physical activity [7, 9] and to a minor extent ill health [10]. Poor health may also lead to poorer education and, thus, hinder occupational attainment [26]. Health-related selection may hence play a role in formation of the occupational class differences in sickness absence [16]. High education provides also knowledge and skills, thus enabling to make better choices in order to promote health [26]. Similarly poor health may lead to poor income and, thus, hinder access to health services and health promotive resources, such as good quality food and leisure time activities [26].

Although several studies have examined occupational class differences in diagnostic-specific sickness absence and explanatory factors to the class disparities, few have focused on changes in occupational class differences in diagnostic-specific sickness absence over time. Currently, several member countries of the Organisation for Economic Co-operation and Development (OECD) consider extension of working lives, for instance by reducing sickness absence, a key target due to ageing workforce [27]. In Finland, various legislative amendments were executed in the early 2010s in order to prevent work disability and employees’ permanent exit from labour market [28]. Diagnostic-specific information on occupational class differences in sickness absence could improve the identification of high-risk groups in terms of work disability, the detection of potential changes in these groups and the targeting of preventive measures effectively in the future [29]. Moreover, the assessment of health and work-life policy interventions calls for monitoring longitudinal trends in socioeconomic differences in health [30]. Reducing socioeconomic inequalities in health has been a key goal in many Finnish health policy programs over the years [31]. During the past decades, socioeconomic health inequalities have remained large in Finland as well as in many European countries [32]. However, little is known about trends over time in diagnostic-specific sickness absence.

Our aim was to examine the magnitude of and changes over time in occupational class differences in long-term sickness absence due to major diagnostic causes, focusing on Finnish women and men during the period 2005–2014. We assessed class differences by means of both absolute and relative measures. This method has been used infrequently in previous studies [33], despite the recommendation of the World Health Organization’s Commission on Social Determinants of Health to use both scales for monitoring socioeconomic inequalities in health over time, thereby giving a more precise picture of the absolute and relative differences [34].

## Methods

### Data

This study is based on a nationally representative 70-per-cent random sample of Finnish residents aged 25–64 years covering the period 2004–2013, and derived from the register of the Social Insurance Institution of Finland (Kela). The sample data constituted an unbalanced panel [35]: in other words individuals could be included in the sample each year between 2004 and 2013, or they could move in and out of the data set. The number of individuals per year may thus vary. In this study, the inclusion in the data set was conditional on individuals’ age, migration and mortality. Overall, the sample data used in this study is representative of Finnish residents aged 25–64 at the end of each year from 2004 to 2013.

The focus was on medically certified long-term sickness absence measured in accordance with information on sickness allowance administered by Kela. Sickness allowance episodes from 2005 to 2014 were derived from the national registers of sickness insurance administered by Kela, including information on paid sickness allowances of all Finnish residents. The data on sickness allowance episodes retrieved from the national registers included the beginning and ending dates, and diagnoses based on the International Classification of Diseases (ICD-10). Under the Finnish system, 16–67-year-olds are eligible for sickness allowance to compensate for work disability caused by an illness or by a home and leisure injury up to approximately 1 year. Work-related and traffic injuries are compensated by insurance companies on the basis of statutory occupational accident insurance and traffic insurance and, thus are not included in this study. Sickness allowance is granted by Kela after a waiting period consisting of the first day of work disability and the following nine working days, i.e. calendar days excluding Sundays and midweek holidays. During the waiting period, work-disabled employees are entitled to full salary paid by employers under the Employment Contracts Act. The waiting period is 55 calendar days for those who have not been employed or engaged in any other gainful activity 3 months prior to the incidence of work disability (this prerequisite expired at the end of 2015), or if annual earned income falls below the minimum level [36]. Long-term sickness absence in this study is defined according to the receipt of sickness allowance (at least one allowance day), and thus refers to absence episodes lasting longer than 10 working days.

The outcome was the receipt of sickness allowance based on major diagnostic causes and any diagnostic cause during a calendar year. The diagnostic causes were categorised according to the main chapters of the ICD-10 [37]: musculoskeletal diseases (M00–M99), mental disorders (F00–F99), injuries (S00–T98), neoplasms (C00–D48), diseases of the nervous system (G00–G99), cardiovascular diseases (I00–I99), respiratory diseases (J00–J99), diseases of the digestive system (K00–K93), other diagnoses (all other ICD-10 diagnosis codes in the data) and any diagnostic cause (any of the ICD-10 diagnosis codes in the data). The three most common diagnostic causes in Finland are musculoskeletal diseases, mental disorders, and injuries [5, 38], other major diagnostic causes being neoplasms, cardiovascular diseases, diseases of the nervous system, diseases of the digestive system and respiratory diseases [38].

Year-end data on occupational class were derived from the register of Statistics Finland and linked to the other data. The categorisation of occupational classes was based on the socio-economic classification of Statistics Finland, comprising seven different categories (see [39] for more details). We focused on three hierarchical occupational classes: upper non-manual employees, lower non-manual employees and manual workers. Old-age pensioners and disability pensioners were excluded from the analysis because they are not entitled to sickness allowance. We also excluded students, the unemployed, and entrepreneurs and farmers. The population at risk of sickness allowance for a given calendar year comprised 25–64-year-old women (yearly n between 658,148 and 694,142) and men (yearly n between 604,715 and 642,922) in each selected hierarchical occupational class at the end of the preceding year. After exclusions, approximately 66–69% of women and 60–63% of men from the original random sample at the end of a year were included in the study population. In total, the dataset comprised 1,930,568 different persons over the study period.

### Statistical methods

The annual age-adjusted prevalence of sickness absence due to the three most common diagnostic causes (musculoskeletal diseases, mental disorders and injuries) by occupational class was calculated from 2005 to 2014. Age was directly standardised using 5-year age groups, with 2010 as the standard population. Age-adjusted prevalence was presented as a percentage with 95-per-cent confidence intervals (CI). All the analyses were conducted separately for women and men.

Linear trend in sickness absence prevalence due to different diagnostic causes by occupational class was examined on data with all years 2005–2014 pooled. This was done for each occupational class separately by including sickness absence as a dependent variable and calendar year as a continuous independent variable to the binomial models, with an identity link function. In addition, the models were further adjusted for age for musculoskeletal diseases, mental disorders and injuries by entering age as an independent variable into the models. Age was classified into 5-year age groups.

Two measures, the Slope Index of Inequality (SII) and the Relative Index of Inequality (RII), were used to the magnitude of absolute and relative occupational class differences in sickness absence due to different diagnostic causes and any cause. The SII and the RII are regression-based summary indices that are recommended for comparing the magnitude of socioeconomic inequalities over time [30]. Both simultaneously take into account potential changes over time in the size and relative socioeconomic position of the groups that are compared. The first step in calculating the indices [40, 41] was to convert the occupational class variable into a relative rank indicator. This was done by ordering the occupational classes from highest to lowest and then transforming the occupational class variable into a relative rank indicator by calculating the midpoint of the range of each occupational class in the cumulative distribution. For example, if upper non-manual employees comprise 30% of women during a given study year, the relative rank indicator for this occupational class would take the value of 0.15 (0.30/2). Correspondingly, if lower non-manuals comprise 50% of the study population among women, the rank indicator would be 0.55 (0.30 + 0.50/2). The rank indicator could take values from 0 (the theoretical top of the class hierarchy) to 1.0 (the theoretical bottom of the hierarchy).

The relative rank indicator was subsequently used as a continuous independent variable in the binomial models, with an identity link function when calculating SII and a log-link function for RII [40, 41]. The SII can be interpreted as the rate difference of having sickness absence between the hypothetical top and the hypothetical bottom of the hierarchy. SII values above 0 indicate higher and values below 0 lower levels of sickness absence in lower compared to higher occupational classes. The RII can be interpreted as the rate ratio of having sickness absence between the hypothetical top and the hypothetical bottom of the hierarchy. RII values above 1.0 indicate higher sickness absence in lower occupational classes and values below 1.0 the reverse. Age-adjusted SII and RII values and confidence intervals (95% CI) were presented for the years 2005, 2008, 2011 and 2014, i.e. each year being a cross-section with regard to time. Age was adjusted using 5-year age groups.

Calendar year and an interaction term of the rank indicator and calendar year were included in the aforementioned models to test for linear trend in absolute (SII) and relative (RII) occupational class differences over time on data with all years 2005–2014 pooled. Due to the size of the data set, some of the changes in the class differences in sickness absence due to different diseases appeared statistically significant but were minor and, thus, had no practical consequence.

SAS statistical software version 9.4 was used to conduct the analyses.

### Results

Table 1 gives the occupational class distributions of the study population for the years 2005, 2008, 2011 and 2014. Non-manual employees comprised the largest occupational class among women, and manual workers among men. During the 10-year study period, the proportions of non-manual workers increased and of manual workers decreased among both women and men.

**Table 1:** Descriptive statistics of the study population in 2005, 2008, 2011 and 2014 ^a^

	2005		2008		2011		2014	
	*n*	%	*n*	%	*n*	%	*n*	%
Women, 25–64 years								
Upper non-manual	149,783	23	163,742	24	165,790	24	170,860	25
Lower non-manual	352,058	53	366,483	53	383,892	56	376,383	56
Manual workers	156,307	24	154,559	23	139,015	20	128,393	19
All	658,148	100	684,784	100	688,697	100	675,636	100
Men, 25–64 years								
Upper non-manual	160,443	26	176,312	27	170,807	27	168,209	28
Lower non-manual	157,726	25	159,377	25	167,233	27	160,297	26
Manual workers	300,198	49	311,037	48	288,682	46	276,209	46
All	618,367	100	646,726	100	626,722	100	604,715	100

^a^ The study population for each year is equal to the population at the end of the preceding year

The overall prevalence of sickness absence due to any diagnostic cause declined between 2005 and 2014 (Table 2). Musculoskeletal diseases were the most common diagnostic cause of long-term sickness absence in the study population. Among women, the second and third most common causes were mental disorders and injuries, respectively. Injuries constituted a more common cause of sickness absence among men than mental disorders. The proportions of both women and men with long-term sickness absence in other diagnostic categories were small, up to 1 %, throughout the study period, and both female and male manual workers had more sickness absence due to any diagnostic cause than their counterparts in higher occupational classes.

**Table 2:** Proportions of persons with long-term sickness absence by diagnostic cause and occupational class ^a^

	Women, 25–64 years				*p* for trend﻿^a﻿^	Men, 25–64 years				*p* for trend^a^
	2005	2008	2011	2014		2005	2008	2011	2014	
	Any cause									
Upper non-manual	11.3	10.9	10.4	9.6	<0.0001	7.0	6.7	6.2	5.8	<0.0001
Lower non-manual	17.0	16.7	16.5	15.5	<0.0001	10.2	10.0	9.8	9.0	<0.0001
Manual workers	21.4	20.3	19.5	18.0	<0.0001	16.3	15.4	14.4	13.2	<0.0001
All	16.7	16.1	15.6	14.5	<0.0001	12.3	11.7	10.9	10.0	<0.0001
	Musculoskeletal									
Upper non-manual	2.5	2.4	2.4	2.1	<0.0001	1.5	1.7	1.5	1.4	<0.0001
Lower non-manual	5.8	5.9	5.8	5.3	<0.0001	3.0	3.1	3.1	2.7	<0.0001
Manual workers	9.9	9.5	9.3	8.3	<0.0001	7.1	6.8	6.2	5.5	<0.0001
All	6.0	5.9	5.6	5.0	<0.0001	4.6	4.5	4.1	3.6	<0.0001
	Mental									
Upper non-manual	2.9	2.8	2.4	2.3	<0.0001	1.4	1.4	1.1	1.0	<0.0001
Lower non-manual	3.5	3.5	3.2	3.1	<0.0001	1.8	1.9	1.6	1.5	<0.0001
Manual workers	3.3	3.2	2.7	2.6	<0.0001	1.7	1.6	1.3	1.2	<0.0001
All	3.3	3.3	2.9	2.8	<0.0001	1.6	1.6	1.3	1.2	<0.0001
	Injuries									
Upper non-manual	1.2	1.2	1.2	1.2	<0.0001	1.2	1.2	1.2	1.2	0.3685
Lower non-manual	1.8	1.9	1.9	1.9	<0.0001	2.0	2.0	2.0	2.0	0.1881
Manual workers	2.3	2.3	2.3	2.2	0.4498	3.3	3.1	3.0	2.8	<0.0001
All	1.8	1.8	1.8	1.8	<0.0001	2.4	2.3	2.3	2.1	<0.0001
	Neoplasms									
Upper non-manual	0.9	0.9	0.9	0.9	0.3528	0.3	0.3	0.4	0.3	0.0123
Lower non-manual	1.0	0.9	0.9	0.9	0.9813	0.3	0.4	0.4	0.4	0.3342
Manual workers	0.9	0.9	0.9	0.9	0.0060	0.4	0.4	0.4	0.4	0.6487
All	0.9	0.9	0.9	0.9	0.0946	0.4	0.4	0.4	0.4	0.0767
	Nervous system									
Upper non-manual	0.4	0.4	0.4	0.4	0.6188	0.2	0.2	0.2	0.2	0.1677
Lower non-manual	0.7	0.7	0.7	0.7	0.0013	0.4	0.4	0.3	0.3	0.0335
Manual workers	1.3	1.2	1.2	1.1	<0.0001	0.7	0.6	0.6	0.7	0.0044
All	0.8	0.8	0.8	0.7	<0.0001	0.5	0.5	0.4	0.4	<0.0001
	Cardiovascular									
Upper non-manual	0.6	0.5	0.4	0.3	<0.0001	0.7	0.6	0.5	0.5	<0.0001
Lower non-manual	0.9	0.7	0.6	0.5	<0.0001	0.8	0.7	0.6	0.5	<0.0001
Manual workers	1.1	0.9	0.7	0.7	<0.0001	1.1	1.0	0.9	0.8	<0.0001
All	0.9	0.7	0.6	0.5	<0.0001	0.9	0.8	0.7	0.6	<0.0001
	Respiratory									
Upper non-manual	0.8	0.8	1.1	0.8	0.1524	0.5	0.4	0.5	0.4	<0.0001
Lower non-manual	1.1	1.0	1.4	1.0	0.1071	0.7	0.6	0.7	0.5	0.0301
Manual workers	1.2	1.0	1.2	0.9	<0.0001	0.9	0.8	0.9	0.6	<0.0001
All	1.1	1.0	1.2	0.9	0.1951	0.7	0.6	0.7	0.5	<0.0001
	Digestive system									
Upper non-manual	0.5	0.5	0.5	0.4	0.0004	0.7	0.6	0.5	0.5	<0.0001
Lower non-manual	0.7	0.6	0.6	0.6	0.0458	0.8	0.7	0.7	0.7	<0.0001
Manual workers	0.7	0.6	0.6	0.6	<0.0001	1.1	0.9	0.9	0.9	<0.0001
All	0.7	0.6	0.6	0.6	<0.0001	0.9	0.8	0.8	0.7	<0.0001
	Other diagnoses									
Upper non-manual	2.4	2.1	2.0	1.8	<0.0001	0.8	0.7	0.7	0.6	<0.0001
Lower non-manual	3.1	2.9	2.8	2.7	<0.0001	1.1	1.0	0.9	0.9	<0.0001
Manual workers	3.1	2.8	2.6	2.5	<0.0001	1.6	1.5	1.3	1.2	<0.0001
All	2.9	2.7	2.6	2.4	<0.0001	1.3	1.1	1.0	0.9	<0.0001

^a^ Unadjusted

**Fig. 1:**
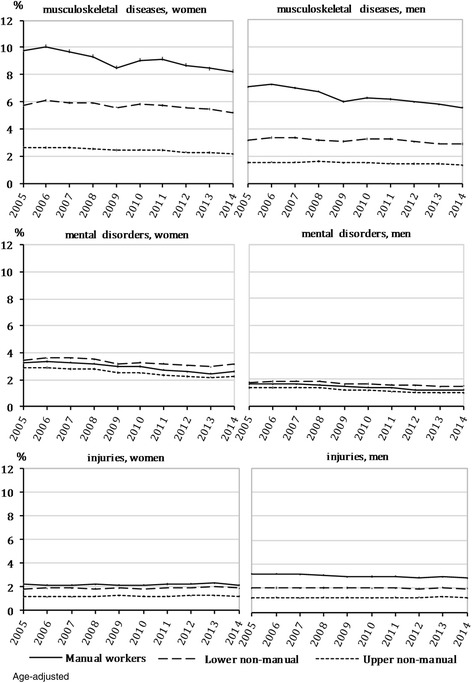
Prevalence of long-term sickness absence due to major diagnostic causes by occupational class, 2005–2014^1^

Among the three most common diagnostic causes of long-term sickness absence, clear hierarchical occupational class differences in age-adjusted prevalence were found for musculoskeletal diseases and injuries in 2005–2014 (Fig. 1). Over the study period, the largest decrease in age-adjusted prevalence due to musculoskeletal diseases occurred among manual workers of both genders (p for trend <0.0001). Among men, the decline in age-adjusted prevalence due to injuries was also most profound among manual workers (p for trend <0.0001), whereas with regard to mental disorders the highest age-adjusted prevalence, and the smallest decrease in age-adjusted prevalence over the study period occurred among the lower non-manuals among both genders (p for trend <0.0001).

The magnitude of and changes in absolute and relative occupational class differences in long-term sickness absence due to diagnostic causes among women.

Absolute differences in long-term sickness absence due to any diagnostic cause measured by the SII were clear in women (Table 3). However, there was a modest declining tendency over time (*p* < 0.0001). Throughout the study period the absolute differences were largest for musculoskeletal diseases: the age-adjusted SII declined between 2005 (SII 0.08, 95% CI 0.07–0.08) and 2014 (SII 0.07, 95% CI 0.06–0.07) (*p* < 0.0001). There were similar modest and stable absolute differences with regard to mental disorders and injuries. Of the remaining diagnostic categories, absolute differences measured by the SII were found in diseases of the nervous system, with no major changes over time, otherwise they remained non-existent.

**Table 3:** Absolute (SII) and relative (RII) occupational class differences in sickness absence by diagnostic causes, women ^a^

Diseases	2005	2008	2011	2014	p for trend
SII ^b^					
Any cause	0.12 (0.12, 0.13)	0.12 (0.11, 0.12)	0.12 (0.12, 0.12)	0.11 (0.11, 0.12)	<0.0001
Musculoskeletal	0.08 (0.07, 0.08)	0.08 (0.07, 0.08)	0.07 (0.07, 0.07)	0.07 (0.06, 0.07)	<0.0001
Mental	0.01 (0.00, 0.01)	0.01 (0.00, 0.01)	0.01 (0.01, 0.01)	0.01 (0.01, 0.01)	0.5135
Injuries	0.01 (0.01, 0.01)	0.01 (0.01, 0.01)	0.01 (0.01, 0.02)	0.01 (0.01, 0.01)	0.8005
Neoplasms	0.00 (0.00, 0.00)	0.00 (0.00, 0.00)	0.00 (0.00, 0.00)	0.00 (0.00, 0.00)	0.4891
Nervous system	0.01 (0.01, 0.01)	0.01 (0.01, 0.01)	0.01 (0.01, 0.01)	0.01 (0.01, 0.01)	<0.0001
Cardiovascular	0.00 (0.00, 0.01)	0.00 (0.00, 0.00)	0.00 (0.00, 0.00)	0.00 (0.00, 0.00)	<0.0001
Respiratory	0.00 (0.00, 0.01)	0.00 (0.00, 0.00)	0.00 (0.00, 0.00)	0.00 (0.00, 0.00)	<0.0001
Digestive system	0.00 (0.00, 0.00)	0.00 (0.00, 0.00)	0.00 (0.00, 0.00)	0.00 (0.00, 0.00)	0.6261
Other diagnoses	0.01 (0.01, 0.01)	0.01 (0.01, 0.01)	0.01 (0.01, 0.01)	0.01 (0.01, 0.01)	0.9142
RII ^b^					
Any cause	2.12 (2.08, 2.16)	2.08 (2.04, 2.13)	2.09 (2.05, 2.14)	2.12 (2.08, 2.17)	0.8737
Musculoskeletal	4.99 (4.80, 5.18)	4.67 (4.50, 4.84)	4.67 (4.50, 4.85)	4.88 (4.68, 5.08)	0.0570
Mental	1.18 (1.12, 1.24)	1.19 (1.14, 1.25)	1.24 (1.18, 1.31)	1.26 (1.19, 1.33)	0.2494
Injuries	2.21 (2.07, 2.37)	2.08 (1.95, 2.28)	2.08 (1.94, 2.22)	2.04 (1.91, 2.19)	0.2226
Neoplasms	1.00 (0.91, 1.10)	0.89 (0.81, 0.98)	0.98 (0.89, 1.08)	0.96 (0.87, 1.05)	0.8106
Nervous system	4.70 (4.23, 5.23)	4.08 (3.67, 4.54)	3.87 (3.47, 4.31)	3.62 (3.24, 4.05)	0.0002
Cardiovascular	2.10 (1.90, 2.32)	2.13 (1.90, 2.37)	2.00 (1.77, 2.25)	2.59 (2.27, 2.96)	0.0227
Respiratory	1.49 (1.36, 1.63)	1.34 (1.22, 1.47)	1.18 (1.08, 1.28)	1.15 (1.04, 1.27)	<0.0001
Digestive system	1.43 (1.27, 1.60)	1.46 (1.29, 1.64)	1.42 (1.26, 1.60)	1.56 (1.38, 1.76)	0.6433
Other diagnoses	1.38 (1.31, 1.45)	1.43 (1.36, 1.52)	1.44 (1.36, 1.52)	1.53 (1.44, 1.62)	0.0042

95% confidence intervals are shown in parentheses

^a^ SII Slope Index of Inequality, RII Relative Index of Inequality

^b^ Age-adjusted; age was classified into 5-year age groups

Women exhibited clear and stable relative differences in long-term sickness absence due to any diagnostic cause measured by the RII (Table 3). Throughout, the largest relative differences were in musculoskeletal diseases: the age-adjusted RII was slightly lower in 2014 (RII 4.88, 95% CI 4.68–5.08) than in 2005 (RII 4.99, 95% CI 4.80–5.18), although the test for linear trend did not confirm statistically significant changes over time (*p* = 0.0570). The relative differences in mental disorders and injuries remained modest and stable over time. With regard to the remaining diagnostic categories, the relative differences were largest in diseases of the nervous system, with a declining trend over time (*p* = 0.0002): the RII was 29-per-cent smaller in 2014 (3.62, 95% CI 3.24–4.05) than in 2005 (4.70, 95% CI 4.23–5.23). The test for linear trend also indicated narrowing relative differences in respiratory diseases (*p* < 0.0001), although the magnitude remained modest throughout the study period. Conversely, the relative differences in cardiovascular diseases tended to widen over time (*p* = 0.0227), resulting in the third highest RII by 2014 (RII 2.59, 95% CI 2.27–2.96) among the diagnostic causes under scrutiny. On the other hand, modest and stable relative differences were found in digestive diseases. Among women, the smallest relative differences throughout the study period were in neoplasms.

The magnitude of and changes in occupational class differences in long-term sickness absence due to diagnostic causes among men.

Men also exhibited clear absolute differences in long-term sickness absence due to any diagnostic cause measured by the SII (Table 4), which narrowed over the study period (*p* < 0.0001). Reflecting the results concerning women, the largest absolute differences were in musculoskeletal diseases: the SII decreased between 2005 (SII 0.08, 95% CI 0.07–0.08) and 2014 (SII 0.06, 95% CI 0.05–0.06) (*p* < 0.0001). With regard to mental disorders, however, the SII revealed no absolute differences among men. The magnitude of absolute differences in injuries was second largest among the diagnostic causes under scrutiny, and the differences remained stable over time. There were modest or non-existent absolute differences in the remaining diagnostic categories: modest differences were found in diseases of the nervous system, respiratory diseases and diseases of the digestive system. There were no major changes in the differences over time in diseases of the nervous and digestive system, whereas there was a narrowing tendency over time in respiratory diseases (*p* < 0.0001).

**Table 4:** Absolute (SII) and relative (RII) occupational class differences in sickness absence by diagnostic causes, men ^a^

Diseases	2005	2008	2011	2014	p for trend
SII ^b^					
Any cause	0.14 (0.14, 0.15)	0.13 (0.13, 0.14)	0.12 (0.12, 0.13)	0.11 (0.11, 0.12)	<0.0001
Musculoskeletal	0.08 (0.07, 0.08)	0.07 (0.07, 0.07)	0.06 (0.06, 0.06)	0.06 (0.05, 0.06)	<0.0001
Mental	0.00 (0.00, 0.01)	0.00 (0.00, 0.00)	0.00 (0.00, 0.00)	0.00 (0.00, 0.00)	0.1907
Injuries	0.03 (0.03, 0.03)	0.03 (0.03, 0.03)	0.03 (0.03, 0.03)	0.03 (0.02, 0.03)	<0.0001
Neoplasms	0.00 (0.00, 0.00)	0.00 (0.00, 0.00)	0.00 (0.00, 0.00)	0.00 (0.00, 0.00)	0.1079
Nervous system	0.01 (0.01, 0.01)	0.01 (0.01, 0.01)	0.01 (0.01, 0.01)	0.01 (0.01, 0.01)	0.0032
Cardiovascular	0.00 (0.00, 0.00)	0.00 (0.00, 0.00)	0.00 (0.00, 0.00)	0.00 (0.00, 0.00)	<0.0001
Respiratory	0.01 (0.01, 0.01)	0.01 (0.00, 0.01)	0.01 (0.01, 0.01)	0.00 (0.00, 0.00)	<0.0001
Digestive system	0.01 (0.01, 0.01)	0.01 (0.00, 0.01)	0.01 (0.00, 0.01)	0.01 (0.01, 0.01)	0.2039
Other diagnoses	0.01 (0.01, 0.01)	0.01 (0.01, 0.01)	0.01 (0.01, 0.01)	0.01 (0.01, 0.01)	<0.0001
RII ^b^					
Any cause	3.76 (3.66, 3.87)	3.62 (3.52, 3.72)	3.51 (3.41, 3.61)	3.51 (3.40, 3.62)	<0.0001
Musculoskeletal	10.77 (10.20, 11.37)	9.27 (8.82, 9.80)	8.33 (7.89, 8.79)	8.54 (8.06, 9.05)	<0.0001
Mental	1.22 (1.13, 1.31)	1.13 (1.06, 1.22)	1.22 (1.13, 1.32)	1.22 (1.12, 1.33)	0.7980
Injuries	4.46 (4.17, 4.77)	4.07 (3.81, 4.35)	3.83 (3.59, 4.10)	3.53 (3.29, 3.78)	<0.0001
Neoplasms	1.66 (1.42, 1.94)	1.42 (1.22, 1.65)	1.41 (1.22, 1.64)	1.39 (1.19, 1.62)	0.0081
Nervous system	5.95 (5.08, 6.97)	5.62 (4.81, 6.56)	6.23 (5.31, 7.32)	6.55 (5.57, 7.71)	0.6117
Cardiovascular	2.45 (2.21, 2.71)	2.59 (2.32, 2.88)	2.62 (2.34, 2.94)	2.62 (2.32, 2.96)	0.3322
Respiratory	2.65 (2.36, 2.98)	2.63 (2.33, 2.98)	2.35 (2.10, 2.63)	2.21 (1.93, 2.53)	0.0329
Digestive system	1.95 (1.76, 2.16)	2.14 (1.92, 2.38)	2.16 (1.94, 2.42)	2.38 (2.12, 2.67)	0.0125
Other diagnoses	2.92 (2.67, 3.19)	3.07 (2.80, 3.36)	2.78 (2.52, 3.01)	2.66 (2.41, 2.95)	0.5200

95% confidence intervals are shown in parentheses

^a^ SII Slope Index of Inequality, RII Relative Index of Inequality

^b^ Age-adjusted; age was classified into 5-year age groups

Men also exhibited clear relative differences in long-term sickness absence due to any diagnostic cause measured by the RII (Table 4), although in contrast to women these relative differences showed a declining trend over time (*p* < 0.0001). Throughout the study period, relative differences were by far the largest in musculoskeletal diseases: although the age-adjusted RII declined by 23% between 2005 (RII 10.77, 95% CI 10.20–11.37) and 2014 (RII 8.54, 95% CI 8.06–9.05) (*p* < 0.0001), the relative differences remained at an exceptionally high level throughout. Conversely, the smallest relative differences were found in mental disorders, and there were no major changes. With regard to injuries, the relative differences tended to narrow over time (*p* < 0.0001) but still remained third largest among the diagnostic causes under scrutiny throughout the study period. Among the remaining diagnostic categories, clear and stable relative differences appeared in diseases of the nervous system. As with women, relative differences in respiratory diseases showed a narrowing tendency over time among men (*p* = 0.0329). On the other hand, relative differences in digestive diseases increased over the study period, and the test for linear trend confirmed a statistically significant change (*p* = 0.0125). With regard to cardiovascular diseases, there were no statistically significant changes in relative differences among men (*p* = 0.3322). In the case of neoplasms, the differences remained second smallest throughout the study period among the diagnostic causes under scrutiny.

## Discussion

This study examined the magnitude of and changes over time in absolute and relative occupational class differences in long-term sickness absence due to major diagnostic causes among Finnish women and men. The large nation-wide data set comprised approximately 1.2–1.3 million persons annually between 2005 and 2014. The three most common diagnostic causes of absence were musculoskeletal diseases, mental disorders and injuries. The prevalence in the other diagnostic categories was low, at most approximately 1 %.

The main findings could be summarised thus. 1) Occupational class differences were by far the largest in the case of long-term sickness absence due to musculoskeletal diseases among both women and men. The relative class differences were particularly large among men throughout the study period. The absolute differences in both genders and the relative differences among men narrowed over time, the prevalence of absences thus attributable declining most rapidly among manual workers. 2) Occupational class differences in sickness absence due to mental disorders were small. Absolute differences were non-existent among men and modest among women, and there were no significant changes over time in relative differences. 3) With regard to injuries, there were stable absolute class differences: among men the relative differences tended to narrow over time in that the prevalence of sickness absence declined most among manual workers. 4) As far as the other diagnostic causes under scrutiny were concerned, there were rather large relative occupational class differences in some cases, such as in diseases of the nervous system, but in absolute terms the class differences appeared negligible throughout the study period.

Our results were consistent with those reported in previous studies showing large occupational class differences in sickness absence due to musculoskeletal diseases [16, 19–21]. A large part of the socioeconomic gradient could well be attributable to differences in health, health behaviours and working conditions, which tend to be more detrimental in manual occupations. Heavy physical work demands, uncomfortable working positions, job dissatisfaction and work stress, for example, have been shown to increase the risk of sickness absence due to musculoskeletal diseases [42, 43]: it was found in a French study [16] that physical and psychosocial work-related factors explained almost half of sickness absence on such grounds among men, and nearly one third among women. Part of the class differences in sickness absence due to musculoskeletal diseases could be attributed to health behaviours [7, 9]; excess weight and smoking, for instance, constitute important risk factors for several musculoskeletal diseases [44]. Overall, higher musculoskeletal morbidity in lower occupational classes [45] also play a role in the formation of the class differences in sickness absence due to musculoskeletal diseases in a working population.

According to our results, both absolute and relative occupational class differences in sickness absence due to musculoskeletal diseases narrowed over time among men, and there was also a declining trend in absolute differences among women. The prevalence decreased in all occupational classes, but in particular among manual workers. The alleviation of physical work demands, for instance as a consequence of increased mechanisation of work, in recent years could explain part of the change [46]. Finnish employees have also reported improvements in occupational safety and health [46]. On the other hand, job insecurity caused by the economic downturn since 2008 may have led to a decline in sickness absence [13, 47], in particular among employees in lower occupational positions [48]. Despite the narrowing trend, however, class differences in sickness absence due to musculoskeletal diseases remained large throughout the study period.

We found that occupational class differences in long-term sickness absence due to mental disorders were at most modest, and remained stable over time. Previous findings on socioeconomic differences in such sickness absence have been mixed, with evidence of a reverse association [15, 24], an inconsistent association [16] and no association for some specific mental diagnoses [25]. In the present study, the proportion of individuals granted sickness absence in these grounds was highest among lower non-manual workers. This occupational class comprises many physically but also mentally demanding occupations (such as nursing, practical nursing, and child-minding). Mentally strenuous working conditions, such as low decision latitude and low social support, have been shown to account for almost 50% of sickness absence related to mental disorders [16]. Less consistent socioeconomic gradients in minor psychiatric disorders [49] may also be reflected in our results. The prevalence of absence on the grounds of mental disorders was fairly low in all the occupational classes under study, however, which could be partially attributable to the health-related selection of employees. Previous studies have shown that poor mental health increases the risk of subsequent unemployment [50] and permanent work disability [51].

Our study revealed clear occupational class differences in sickness absences due to home and leisure injuries, which are the most common types of injury among Finnish working-age people [52]. This finding is in line with the results of previous studies showing hierarchical socioeconomic differences in sickness absence due to work injuries [15–18]. Overall, there are clear socioeconomic differences in the risk of injury in both work-related and non-occupational settings [53], and the main explanations lie in individual and contextual factors [54]. The major contributors to injuries among Finnish working-age population include medication, drugs and alcohol [52]. Unhealthy alcohol drinking habits have been previously shown to increase the risk of medically certified sickness absence [55]. The risk of alcohol-related health consequences tends to be higher among manual workers than among those in higher classes, even with the same consumption levels [56]. These findings may, at least in part, explain our results which could also have been affected by the diverse work-ability requirements in different occupations. In addition, employees in higher occupational classes may have better opportunities to adapt work tasks compared to employees in lower classes. Relative differences tended to narrow among men between 2005 and 2014, however, as the prevalence decreased among manual workers over the study period. One explanation for this change could be the price increase in alcohol following the tightening of taxation, and the consequently reduced alcohol consumption in Finland since 2007 [57]; previous studies have indicated that changes in alcohol prices have biggest effect on alcohol consumption [58] and alcohol-related harm [59] among manual workers, and men in particular.

The prevalence of the other studied diagnostic causes of sickness absence under study was low, and the absolute class differences modest. The relative differences in diseases of nervous system were somewhat large, although they narrowed among women during the study period, the prevalence declining most among manual workers. Socioeconomic differences in morbidity could help to explain class differences in long-term sickness absence attributable to diseases of nervous system [60]: for instance, some manual occupations such as construction workers, dry cleaners and launderers carry an increased risk of hospitalization due to epilepsy, one cause of which is suggested to be frequent exposure to chemicals [61].

The magnitude of occupational class differences in sickness absence varied in the present study depending on the diagnostic cause. This is consistent with the finding of previous studies examining socioeconomic differences in sickness absence simultaneously across various disease categories [15, 16]. A medical diagnosis is a prerequisite for prolonged absence from work, and a certified sickness absence is granted only if a disease leads to an imbalance between work ability and demands [3]. Our results, in line with those reported in earlier studies, imply that the contribution of factors related socioeconomic position, such as ill health, deleterious health behaviours, and physical and psychosocial working conditions, to sickness absence may also differ depending on a disease for sickness absence. Further, our study showed that changes over time in the class differences varied between different diagnostic causes. The class differences have remained relatively stable in several different diagnostic categories over time. Similar trend has been detected previously in both absolute and relative occupational class differences in health in Finland [32]. However, a narrowing trend in the class differences was found in sickness absence attributable to musculoskeletal diseases in the present study. This change is noteworthy since musculoskeletal diseases constitute the single most common diagnostic cause of long-term sickness absence in Finnish working population. The potential for prevention has previously been shown to be particularly high in the case of musculoskeletal diseases [62]. In the future, preventive actions should be continued and targeted particularly to lower occupational classes and to the major diagnostic causes for long-term sickness absence, i.e. musculoskeletal diseases, mental disorders and injuries, when attempting to reduce sickness absence and narrow the class differences.

### Strengths and limitations

This study was based on a nationally representative sample of the Finnish working-age population covering a 10-year period and obtained from a comprehensive national register database. The sample data was linked to register data on sickness absence episodes exceeding 10 working days, with practically no missing information. All such episodes were medically certified, thus eliminating self-report bias. Additionally, the data covered a broad range of diagnoses. Data on occupational class (upper non-manuals, lower non-manuals and manual workers) were retrieved from a national register comprising information from several occupations in different sectors. We used both absolute and relative measures to examine occupational class differences in sickness absence, which is rare done in previous studies. Our results can be directly generalised to the labour force in Finland and with caution to other countries as well with regard to the occupational classes under scrutiny.

The present study also has some limitations. For instance, we were unable to suggest explanations for the class differences in sickness absence attributable to the different diagnostic causes because we lacked national register data on morbidity, health-related behaviours and working conditions. Nation-wide register data cover all sickness absence episodes in Finland lasting longer than 10 working days based on sickness allowance paid by Kela. However, there are no national registers incorporating shorter sickness absence episodes, which could therefore not be included in this study. Short sickness absence is more typical, in cases of respiratory diseases and gastrointestinal infections, for instance, whereas absences tend to be longer in cases of musculoskeletal diseases and mental disorders [15]. A British study [15] examined socioeconomic differences in shorter (7 days or less) periods of sickness absence due to several diagnostic causes, and also reposted particularly large differences in gastrointestinal infections and other diseases of the digestive system. This could have been the case in our study had we included shorter sickness absence episodes in the analyses.

## Conclusions

Several European countries have implemented policy actions to reduce sickness absence, given its considerable economic burden on society. A proposal to implement national guidelines for physicians in Finland, for instance, has been introduced to facilitate estimation of the need for and duration of sickness absence for common diseases and injuries [63]. According to the results of the present study, the most common diagnostic causes of long-term sickness absence among Finnish working-age population are musculoskeletal diseases, mental disorders and injuries. By far the largest occupational class differences in long-term sickness absence were in the prevalence of musculoskeletal diseases, followed by injuries. Our findings highlight the potential targets of preventive measures to tackle socioeconomic differences in sickness absence, and to reduce overall economic burden of work disability on society in the future.

## Abbreviations

CI: Confidence interval
ICD-10: The International Classification of Diseases, 10th revision
Kela: The Social Insurance Institution of Finland
RII: Relative Index of Inequality
SII: Slope Index of Inequality
